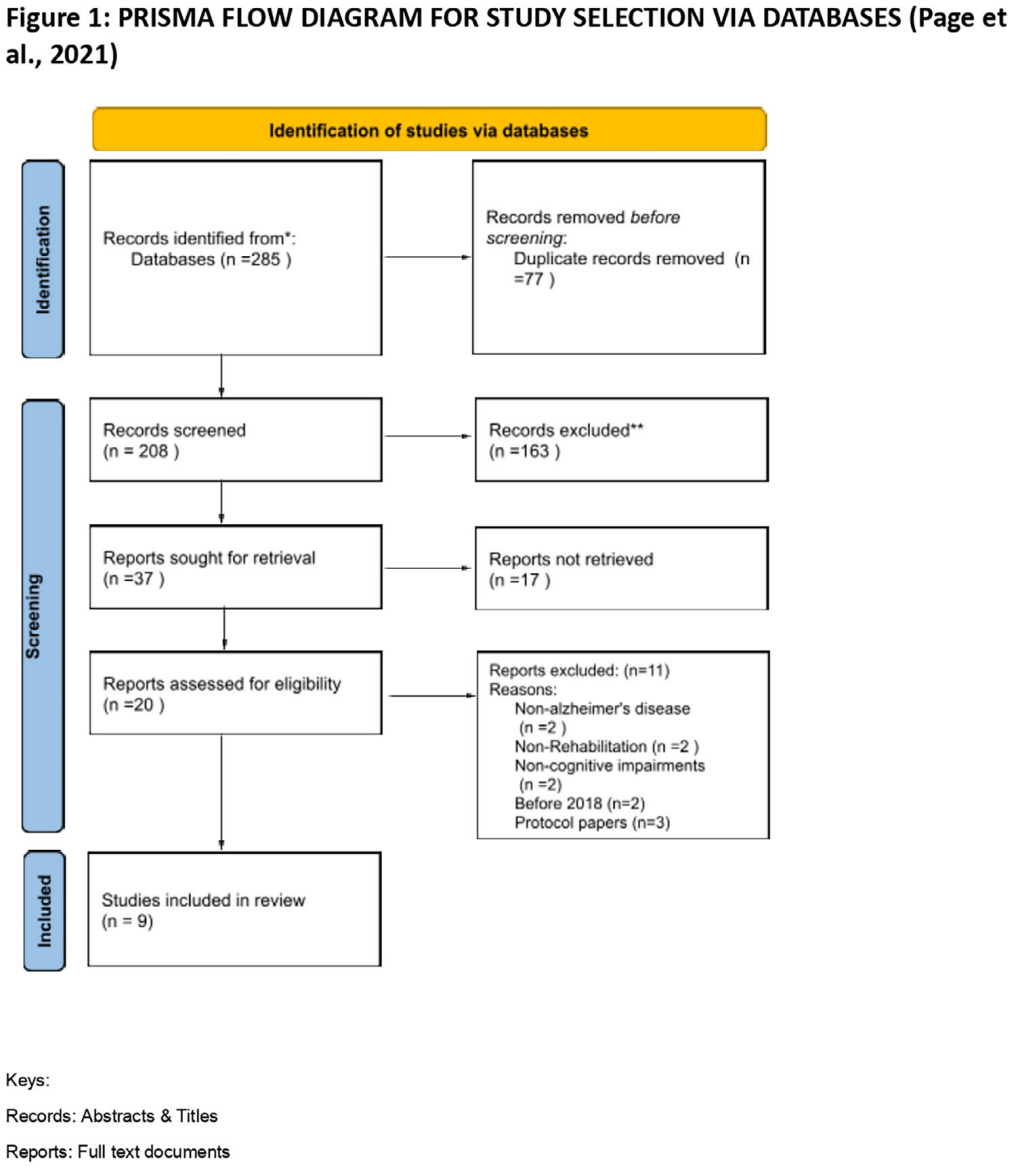# Telerehabilitation Interventions in the management of Alzheimer’s disease: An Implication for rehabilitation care in Sub‐Saharan Africa

**DOI:** 10.1002/alz.086775

**Published:** 2025-01-09

**Authors:** Mary Akinwola, Monsurat Oladosu, Olawale O Oladimeji, Joshua Okafor, Isaac Fredrick, Winnifred Omono Okoro, Tobi Adekolurejo

**Affiliations:** ^1^ College of Medicine, University of Ibadan, Ibadan, Oyo Nigeria; ^2^ University of Nigeria, Nsukka, Enugu Nigeria

## Abstract

**Background:**

Alzheimer’s disease is estimated to reach 139 million people by 2050, with an increase in people living with functional limitations caused by the disease (Alzheimer’s Disease International). However, telerehabilitation presents a promising solution to improve functional outcomes in patients with Alzheimer’s disease. This systematic review investigates the use of telerehabilitation therapies worldwide for the treatment of Alzheimer’s disease and cognitive impairments. This study is the first to provide telerehabilitation insights for managing Alzheimer’s disease in Sub‐Saharan African under‐developed countries.

**Method:**

This systematic review examined telerehabilitation, Alzheimer’s, and cognitive impairment in published studies. Keywords were used to search PubMed, Embase, Cochrane Library, PEDro, Science Direct, SpringerLink, and IEEE Xplore. The search was limited to 2018 studies, not areas. For two screening steps, four reviewers extracted the studies into an Excel spreadsheet. RCT bias was examined using the CASP Randomised Controlled Trial Standard Checklist. The Consolidated Framework for Implementation study identified barriers, successes, and recommendations. Finally, two reviewers used narrative synthesis to identify data themes.

**Result:**

Nine published studies were from high‐income countries. In the case of telerehabilitation intervention, video conferencing was found to be the most common (66%). There were a total of 5 RCT studies and all reported improved cognitive and physical functioning outcomes similar to the control groups. The feasibility studies showed that telerehabilitation patients adhered to treatment. Internet accessibility was a key impediment for rural areas receiving home‐based therapies. Direct supervision, individualized exercise prescriptions, and group exercise treatment facilitate remote telerehabilitation.

**Conclusion:**

The use of telerehabilitation improves cognitive and physical results in Alzheimer’s disease and cognitive impairment patients. This study only covered high‐income nations, but it offers insights for developing sub‐Saharan African countries to improve Alzheimer’s disease management via telerehabilitation. This comprehensive study can help Sub‐Saharan African healthcare practitioners establish telerehabilitation programmes for Alzheimer’s disease and cognitive impairment patients to enhance their functional results.